# Diaqua­bis­(*N*,*N*′-diethyl­nicotinamide-κ*N* ^1^)bis­(4-ethyl­benzoato-κ*O*)copper(II)

**DOI:** 10.1107/S1600536811018666

**Published:** 2011-05-20

**Authors:** Hacali Necefoğlu, Ali Maracı, Vedat Aktaş, Barış Tercan, Tuncer Hökelek

**Affiliations:** a Department of Chemistry, Kafkas University, 36100 Kars, Turkey; b Department of Physics, Karabük University, 78050, Karabük, Turkey; c Department of Physics, Hacettepe University, 06800 Beytepe, Ankara, Turkey

## Abstract

The title Cu^II^ complex, [Cu(C_9_H_9_O_2_)_2_(C_10_H_14_N_2_O)_2_(H_2_O)_2_], contains two 4-ethyl­benzoate (PEB) ligands, two monodentate diethyl­nicotinamide (DENA) ligands and two water mol­ecules. The four O atoms in the equatorial plane around the Cu^II^ ion form a slightly distorted square-planar arrangement, while the distorted octa­hedral coordination is completed by the two N atoms of the DENA ligands in the axial positions. Intra­molecular O—H⋯O hydrogen bonds link the water mol­ecules to the carboxyl­ate groups. The dihedral angles between the carboxyl­ate groups and the adjacent benzene rings are 4.6 (3) and 3.7 (2)°, while the pyridine rings and the benzene rings are oriented at dihedral angles of 6.82 (11) and 3.63 (14)°. In the crystal, inter­molecular O—H⋯O hydrogen bonds link the mol­ecules into chains propagating along [010]. C—H⋯O inter­actions and a π–π contact between the pyridine rings [centroid–centroid distance = 3.469 (2) Å] are also observed.

## Related literature

For literature on niacin, see: Krishnamachari (1974 ▶). For information on the nicotinic acid derivative *N*,*N*-diethyl­nicotinamide, see: Bigoli *et al.* (1972 ▶). For related structures, see: Hökelek *et al.* (1996 ▶, 2009*a* ▶,*b* ▶); Hökelek & Necefoğlu (1998 ▶, 2007 ▶); Necefoğlu *et al.* (2011 ▶). For bond-length data, see: Allen *et al.* (1987 ▶).
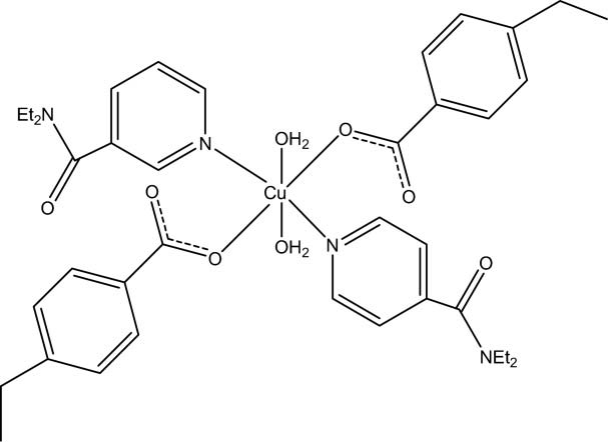


## Experimental

### 

#### Crystal data

[Cu(C_9_H_9_O_2_)_2_(C_10_H_14_N_2_O)_2_(H_2_O)_2_]
*M* *_r_* = 754.37
Monoclinic, 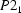

*a* = 8.3607 (2) Å
*b* = 12.4053 (4) Å
*c* = 17.8932 (6) Å
β = 98.132 (3)°
*V* = 1837.17 (10) Å^3^
*Z* = 2
Mo *K*α radiation
μ = 0.65 mm^−1^
*T* = 100 K
0.34 × 0.32 × 0.24 mm

#### Data collection

Bruker Kappa APEXII CCD area-detector diffractometer
18349 measured reflections
8952 independent reflections
6851 reflections with *I* > 2σ(*I*)
*R*_int_ = 0.068

#### Refinement

*R*[*F* ^2^ > 2σ(*F* ^2^)] = 0.056
*wR*(*F* ^2^) = 0.130
*S* = 1.06
8952 reflections
479 parameters
5 restraints
H atoms treated by a mixture of independent and constrained refinement
Δρ_max_ = 0.88 e Å^−3^
Δρ_min_ = −1.09 e Å^−3^
Absolute structure: Flack (1983 ▶), 4105 Friedel pairs
Flack parameter: 0.394 (13)

### 

Data collection: *APEX2* (Bruker, 2007 ▶); cell refinement: *SAINT* (Bruker, 2007 ▶); data reduction: *SAINT*; program(s) used to solve structure: *SHELXS97* (Sheldrick, 2008 ▶); program(s) used to refine structure: *SHELXL97* (Sheldrick, 2008 ▶); molecular graphics: *ORTEP-3 for Windows* (Farrugia, 1997 ▶); software used to prepare material for publication: *WinGX* (Farrugia, 1999 ▶) and *PLATON* (Spek, 2009 ▶).

## Supplementary Material

Crystal structure: contains datablocks I, global. DOI: 10.1107/S1600536811018666/su2274sup1.cif

Structure factors: contains datablocks I. DOI: 10.1107/S1600536811018666/su2274Isup2.hkl

Additional supplementary materials:  crystallographic information; 3D view; checkCIF report

## Figures and Tables

**Table 1:** Hydrogen-bond geometry (Å, °)

*D*—H⋯*A*	*D*—H	H⋯*A*	*D*⋯*A*	*D*—H⋯*A*
O7—H71⋯O2	0.85 (2)	1.86 (2)	2.706 (4)	170 (5)
O7—H72⋯O6^i^	0.83 (4)	2.03 (4)	2.846 (4)	166 (5)
O8—H81⋯O4	0.85 (2)	1.88 (2)	2.699 (4)	161 (5)
O8—H82⋯O2^ii^	0.86 (4)	2.00 (4)	2.852 (4)	167 (5)
C6—H6⋯O5^iii^	0.93	2.55	3.240 (5)	131
C20—H20⋯O2^iii^	0.93	2.53	3.412 (5)	158
C30—H30⋯O6^iv^	0.93	2.43	3.316 (5)	158

Symmetry codes: (i) 
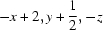
; (ii) 
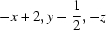
; (iii) 
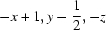
; (iv) 
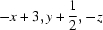
.

**Fig. 1.**
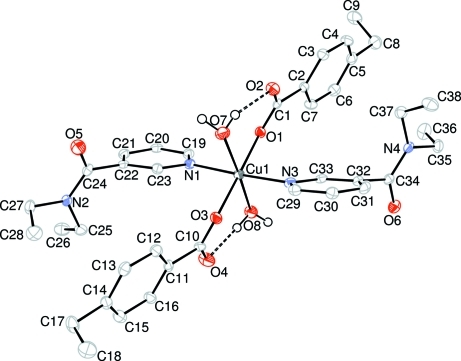
The molecular structure of the title molecule with the atom-numbering scheme. Displacement ellipsoids are drawn at the 50% probability level. The intramolecular O-H···O hydrogen bonds are shown as dashed lines.

**Fig. 2.**
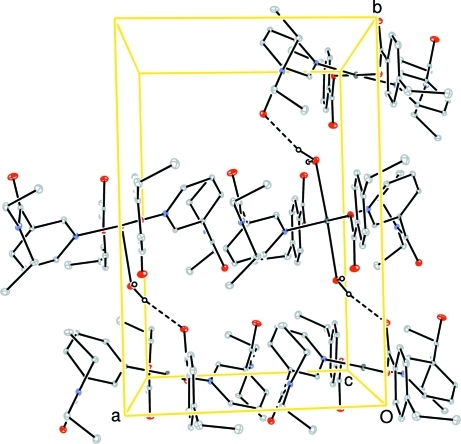
A view along the c-axis of the crystal packing of the title compound. Only the O-H···O hydrogen bonds are shown as dashed lines [H-atoms not involved in hydrogen bonding have been omitted for clarity; see Table 1 for details].

## supplementary crystallographic information

### Comment

As a part of our ongoing investigations of transition metal complexes of nicotinamide (NA), one form of niacin (Krishnamachari, 1974), and/or the nicotinic acid derivative *N*,*N*-diethylnicotinamide (DENA), an important respiratory stimulant (Bigoli *et al.*, 1972), the title compound was synthesized and its crystal structure is reported herein.

The title mononuclear Cu^II^complex, (Fig. 1), consisting of two *N*,*N*-diethylnicotinamide (DENA), two 4-ethylbenzoate (PEB) ligands and two coordinated water molecules, all ligands coordinating in a monodentate manner. The crystal structures of similar omplexes of Cu^II^, Co^II^, Ni^II^, Mn^II^ and Zn^II^ ions, [Cu(C_7_H_5_O_2_)_2_(C_10_H_14_N_2_O)_2_] (Hökelek *et al.*, 1996), [Co(C_6_H_6_N_2_O)_2_(C_7_H_4_NO_4_)_2_(H_2_O)_2_] (Hökelek & Necefoğlu, 1998), [Co(C_9_H_9_O_2_)_2_(C_10_H_14_N_2_O)_2_(H_2_O)_2_] (Necefoğlu *et al.*, 2011), [Ni(C_7_H_4_ClO_2_)_2_(C_6_H_6_N_2_O)_2_(H_2_O)_2_] (Hökelek *et al.*, 2009*a*), [Mn(C_9_H_10_NO_2_)_2_(H_2_O)_4_].2H_2_O (Hökelek & Necefoğlu, 2007) and [Zn(C_7_H_4_BrO_2_)_2_(C_6_H_6_N_2_O)_2_(H_2_O)_2_] (Hökelek *et al.*, 2009*b*) have also been reported. In the copper(II) complex mentioned above the two benzoate ions coordinate to the Cu^II^ atom as bidentate ligands, while in the other structures all the ligands coordinate in a monodentate manner.

In the title complex, the four O atoms (O1, O3, O7 and O8) in the equatorial plane around the Cu^II^ ion form a slightly distorted square-planar arrangement, while the slightly distorted octahedral coordination is completed by the two N atoms of the DENA ligands (N1 and N3) in the axial positions. Intramolecular O-H···O hydrogen bonds link the water molecules to the carboxylate groups (Table 1 and Fig. 1). The near equalities of the C1—O1 [1.275 (4) Å], C1—O2 [1.253 (4) Å] and C10—O3 [1.268 (4) Å], C10—O4 [1.234 (4) Å] bonds in the carboxylate groups indicate delocalized bonding arrangements, rather than localized single and double bonds. The Cu—O bond lengths are 1.968 (2) and 1.979 (2) Å (for benzoate oxygens) and 2.486 (3) and 2.439 (3) Å (for water oxygens), and the Cu—N bond lengths are 2.004 (3) and 2.004 (3) Å, close to standard values (Allen *et al.*, 1987). The Cu atom is displaced out of the mean-planes of the carboxylate groups (O1/C1/O2) and (O3/C10/O4) by -0.7205 (4) and 0.7343 (4) Å, respectively. The dihedral angles between the planar carboxylate groups and the adjacent benzene rings A (C2—C7) and B (C11—C16) are 4.64 (25) and 3.67 (23) °, respectively. The benzene A (C2—C7) and B (C11—C16) rings and the pyridine C (N1/C19—C23) and D (N3/C29—C33) rings are oriented at dihedral angles of A/B = 3.63 (14), A/C = 66.65 (14), A/D = 61.40 (14), B/C = 66.93 (13), B/D = 61.39 (13) and C/D = 6.82 (11) °.

In the crystal, intermolecular O—H···O hydrogen bonds link the molecules into chains propagating along [010] (Table 1 and Fig. 2). There also exist C-H···O interactions leading to the formation of two-dimensional networks lying parallel to (110). The π–π contact between the pyridine rings, Cg3—Cg4^i^, may further stabilize the structure [centroid-centroid distance = 3.469 (2) Å; symmetry code: (i) x - 1, y, z; Cg3 and Cg4 are the centroids of the rings C (N1/C19—C23) and D (N3/C29—C33), respectively].

### Experimental

The title compound was prepared by the reaction of CuSO_4_.5H_2_O (1.23 g, 5 mmol) in H_2_O (100 ml) and DENA (1.78 g, 10 mmol) in H_2_O (50 ml) with sodium 4-ethylbenzoate (1.72 g, 10 mmol) in H_2_O (100 ml) at room temperature. The mixture was filtered and set aside to crystallize at ambient temperature for three days, giving blue single crystals.

### Refinement

The compound crystallized as an inversion twin: refined BASF parameter = 0.394 (13), for 4105 Friedel pairs (84.7% coverage). Atoms H71, H72, H81 and H82 (for water molecules) were located in a difference Fourier map and were freely refined. The C-bound H-atoms were positioned geometrically with C—H = 0.93, 0.97 and 0.96 Å, for aromatic, methylene and methyl H-atoms, respectively, and constrained to ride on their parent atoms, with *U*_iso_(H) = k × *U*_eq_(C), where k = 1.5 for methyl H-atoms and k = 1.2 for all other H-atoms.

### Crystal data

**Table:**

[Cu(C_9_H_9_O_2_)_2_(C_10_H_14_N_2_O)_2_(H_2_O)_2_]	*F*(000) = 798
*M**_r_* = 754.37	*D*_x_ = 1.364 Mg m^−^^3^
Monoclinic, *P*2_1_	Mo *K*α radiation, λ = 0.71073 Å
Hall symbol: P 2yb	Cell parameters from 3109 reflections
*a* = 8.3607 (2) Å	θ = 2.8–26.9°
*b* = 12.4053 (4) Å	µ = 0.65 mm^−^^1^
*c* = 17.8932 (6) Å	*T* = 100 K
β = 98.132 (3)°	Block, blue
*V* = 1837.17 (10) Å^3^	0.34 × 0.32 × 0.24 mm
*Z* = 2	

### Data collection

**Table:**

Bruker Kappa APEXII CCD area-detector diffractometer	6851 reflections with *I* > 2σ(*I*)
Radiation source: fine-focus sealed tube	*R*_int_ = 0.068
graphite	θ_max_ = 28.5°, θ_min_ = 2.3°
φ and ω scans	*h* = −10→11
18349 measured reflections	*k* = −16→16
8952 independent reflections	*l* = −23→20

### Refinement

**Table:**

Refinement on *F*^2^	Secondary atom site location: difference Fourier map
Least-squares matrix: full	Hydrogen site location: inferred from neighbouring sites
*R*[*F*^2^ > 2σ(*F*^2^)] = 0.056	H atoms treated by a mixture of independent and constrained refinement
*wR*(*F*^2^) = 0.130	*w* = 1/[σ^2^(*F*_o_^2^) + (0.0415*P*)^2^ + 0.0395*P*] where *P* = (*F*_o_^2^ + 2*F*_c_^2^)/3
*S* = 1.06	(Δ/σ)_max_ < 0.001
8952 reflections	Δρ_max_ = 0.88 e Å^−^^3^
479 parameters	Δρ_min_ = −1.09 e Å^−^^3^
5 restraints	Absolute structure: Flack (1983), 4105 Friedel pairs
Primary atom site location: structure-invariant direct methods	Flack parameter: 0.394 (13)

### Special details

**Table:**

Geometry. All esds (except the esd in the dihedral angle between two l.s. planes) are estimated using the full covariance matrix. The cell esds are taken into account individually in the estimation of esds in distances, angles and torsion angles; correlations between esds in cell parameters are only used when they are defined by crystal symmetry. An approximate (isotropic) treatment of cell esds is used for estimating esds involving l.s. planes.
Refinement. Refinement of F^2^ against ALL reflections. The weighted R-factor wR and goodness of fit S are based on F^2^, conventional R-factors R are based on F, with F set to zero for negative F^2^. The threshold expression of F^2^ > 2sigma(F^2^) is used only for calculating R-factors(gt) etc. and is not relevant to the choice of reflections for refinement. R-factors based on F^2^ are statistically about twice as large as those based on F, and R- factors based on ALL data will be even larger.

### Fractional atomic coordinates and isotropic or equivalent isotropic displacement parameters (Å^2^)

**Table:**

	*x*	*y*	*z*	*U*_iso_*/*U*_eq_	
Cu1	0.90474 (5)	−0.00022 (4)	0.08866 (2)	0.01551 (11)	
O1	0.8037 (3)	−0.0117 (2)	−0.01725 (13)	0.0158 (5)	
O2	0.8098 (4)	0.1582 (2)	−0.05880 (15)	0.0207 (6)	
O3	1.0046 (3)	0.0129 (2)	0.19537 (13)	0.0185 (6)	
O4	1.0117 (4)	−0.1561 (2)	0.23888 (16)	0.0283 (7)	
O5	0.4397 (4)	0.1513 (2)	0.27561 (17)	0.0295 (7)	
O6	1.3973 (3)	−0.1478 (2)	−0.08725 (15)	0.0217 (6)	
O7	0.8548 (4)	0.1972 (2)	0.09151 (16)	0.0236 (7)	
H71	0.843 (6)	0.193 (4)	0.0436 (11)	0.040*	
H72	0.781 (5)	0.240 (3)	0.098 (3)	0.040*	
O8	0.9451 (4)	−0.1949 (2)	0.08928 (16)	0.0228 (7)	
H81	0.984 (6)	−0.192 (4)	0.1360 (12)	0.040*	
H82	1.012 (5)	−0.239 (3)	0.073 (3)	0.040*	
N1	0.6909 (4)	−0.0310 (2)	0.12257 (17)	0.0154 (7)	
N2	0.4762 (3)	−0.0024 (3)	0.34583 (15)	0.0173 (6)	
N3	1.1134 (4)	0.0359 (2)	0.05150 (17)	0.0156 (7)	
N4	1.3057 (4)	−0.0215 (2)	−0.17411 (17)	0.0159 (7)	
C1	0.8017 (4)	0.0584 (3)	−0.0694 (2)	0.0143 (8)	
C2	0.7873 (4)	0.0155 (3)	−0.1482 (2)	0.0161 (8)	
C3	0.7745 (5)	0.0849 (3)	−0.2100 (2)	0.0193 (9)	
H3	0.7739	0.1591	−0.2028	0.023*	
C4	0.7626 (6)	0.0422 (4)	−0.2826 (3)	0.0224 (10)	
H4	0.7540	0.0886	−0.3238	0.027*	
C5	0.7634 (6)	−0.0679 (4)	−0.2946 (3)	0.0215 (10)	
C6	0.7803 (5)	−0.1364 (3)	−0.2331 (2)	0.0213 (9)	
H6	0.7840	−0.2105	−0.2404	0.026*	
C7	0.7917 (5)	−0.0953 (3)	−0.1607 (2)	0.0187 (9)	
H7	0.8024	−0.1423	−0.1198	0.022*	
C8	0.7400 (5)	−0.1134 (4)	−0.3746 (2)	0.0286 (10)	
H8A	0.7903	−0.0655	−0.4073	0.034*	
H8B	0.7930	−0.1829	−0.3746	0.034*	
C9	0.5623 (5)	−0.1262 (4)	−0.4056 (2)	0.0294 (10)	
H9A	0.5520	−0.1522	−0.4566	0.044*	
H9B	0.5090	−0.0578	−0.4047	0.044*	
H9C	0.5135	−0.1768	−0.3751	0.044*	
C10	1.0113 (5)	−0.0573 (3)	0.2473 (2)	0.0172 (8)	
C11	1.0164 (4)	−0.0133 (4)	0.3264 (2)	0.0163 (8)	
C12	1.0053 (5)	0.0963 (3)	0.3396 (2)	0.0186 (8)	
H12	0.9974	0.1445	0.2994	0.022*	
C13	1.0058 (5)	0.1342 (3)	0.4124 (2)	0.0202 (9)	
H13	0.9958	0.2079	0.4203	0.024*	
C14	1.0208 (5)	0.0653 (3)	0.4734 (2)	0.0193 (9)	
C15	1.0304 (6)	−0.0448 (3)	0.4603 (3)	0.0225 (10)	
H15	1.0389	−0.0927	0.5006	0.027*	
C16	1.0274 (5)	−0.0837 (3)	0.3869 (2)	0.0189 (8)	
H16	1.0328	−0.1575	0.3786	0.023*	
C17	1.0308 (5)	0.1098 (4)	0.5526 (2)	0.0256 (9)	
H17A	0.9463	0.1630	0.5540	0.031*	
H17B	1.0124	0.0518	0.5868	0.031*	
C18	1.1927 (6)	0.1611 (4)	0.5792 (3)	0.0330 (11)	
H18A	1.1941	0.1880	0.6296	0.050*	
H18B	1.2103	0.2196	0.5462	0.050*	
H18C	1.2767	0.1084	0.5787	0.050*	
C19	0.5897 (5)	−0.1049 (3)	0.0867 (2)	0.0159 (8)	
H19	0.6190	−0.1397	0.0446	0.019*	
C20	0.4438 (5)	−0.1308 (3)	0.1102 (2)	0.0173 (8)	
H20	0.3740	−0.1798	0.0832	0.021*	
C21	0.4034 (5)	−0.0825 (3)	0.1749 (2)	0.0177 (8)	
H21	0.3082	−0.1011	0.1930	0.021*	
C22	0.5073 (4)	−0.0054 (4)	0.21259 (18)	0.0148 (7)	
C23	0.6480 (4)	0.0176 (3)	0.18347 (19)	0.0166 (8)	
H23	0.7168	0.0698	0.2076	0.020*	
C24	0.4714 (5)	0.0550 (3)	0.2805 (2)	0.0188 (9)	
C25	0.5422 (5)	−0.1111 (3)	0.3563 (2)	0.0202 (9)	
H25A	0.6213	−0.1126	0.4015	0.024*	
H25B	0.5973	−0.1292	0.3138	0.024*	
C26	0.4135 (5)	−0.1956 (3)	0.3633 (2)	0.0264 (10)	
H26A	0.4633	−0.2653	0.3708	0.040*	
H26B	0.3368	−0.1964	0.3180	0.040*	
H26C	0.3590	−0.1786	0.4056	0.040*	
C27	0.4392 (5)	0.0549 (3)	0.4128 (2)	0.0210 (9)	
H27A	0.3895	0.0053	0.4446	0.025*	
H27B	0.3624	0.1120	0.3974	0.025*	
C28	0.5903 (5)	0.1029 (4)	0.4578 (2)	0.0274 (10)	
H28A	0.5635	0.1342	0.5035	0.041*	
H28B	0.6333	0.1576	0.4283	0.041*	
H28C	0.6695	0.0473	0.4700	0.041*	
C29	1.2063 (5)	0.1172 (3)	0.0815 (2)	0.0167 (8)	
H29	1.1771	0.1534	0.1230	0.020*	
C30	1.3441 (5)	0.1496 (3)	0.0530 (2)	0.0174 (8)	
H30	1.4069	0.2058	0.0755	0.021*	
C31	1.3871 (5)	0.0969 (3)	−0.0093 (2)	0.0182 (9)	
H31	1.4778	0.1183	−0.0301	0.022*	
C32	1.2920 (4)	0.0111 (3)	−0.04045 (19)	0.0152 (7)	
C33	1.1567 (4)	−0.0163 (3)	−0.00806 (19)	0.0150 (8)	
H33	1.0931	−0.0735	−0.0285	0.018*	
C34	1.3359 (5)	−0.0583 (3)	−0.1027 (2)	0.0152 (8)	
C35	1.3510 (5)	−0.0871 (3)	−0.2355 (2)	0.0211 (9)	
H35A	1.4413	−0.1330	−0.2160	0.025*	
H35B	1.3859	−0.0402	−0.2734	0.025*	
C36	1.2124 (5)	−0.1569 (4)	−0.2719 (2)	0.0295 (10)	
H36A	1.2479	−0.1996	−0.3112	0.044*	
H36B	1.1242	−0.1117	−0.2931	0.044*	
H36C	1.1775	−0.2035	−0.2346	0.044*	
C37	1.2226 (5)	0.0805 (3)	−0.1947 (2)	0.0196 (9)	
H37A	1.1667	0.1036	−0.1535	0.024*	
H37B	1.1423	0.0691	−0.2387	0.024*	
C38	1.3381 (6)	0.1688 (3)	−0.2118 (2)	0.0267 (10)	
H38A	1.2784	0.2338	−0.2252	0.040*	
H38B	1.3926	0.1468	−0.2530	0.040*	
H38C	1.4161	0.1817	−0.1680	0.040*	

### Atomic displacement parameters (Å^2^)

**Table:**

	*U*^11^	*U*^22^	*U*^33^	*U*^12^	*U*^13^	*U*^23^
Cu1	0.0124 (2)	0.02013 (19)	0.0137 (2)	−0.0006 (2)	0.00064 (15)	0.00015 (19)
O1	0.0138 (12)	0.0190 (13)	0.0145 (12)	−0.0019 (13)	0.0022 (10)	0.0001 (13)
O2	0.0265 (17)	0.0162 (13)	0.0196 (15)	0.0010 (12)	0.0035 (13)	−0.0011 (11)
O3	0.0157 (13)	0.0243 (15)	0.0154 (12)	−0.0010 (13)	0.0021 (10)	0.0012 (13)
O4	0.042 (2)	0.0207 (15)	0.0213 (16)	0.0028 (14)	0.0005 (15)	−0.0015 (13)
O5	0.043 (2)	0.0183 (14)	0.0282 (17)	0.0087 (14)	0.0101 (15)	0.0005 (12)
O6	0.0207 (16)	0.0195 (13)	0.0252 (16)	0.0023 (12)	0.0047 (13)	−0.0013 (12)
O7	0.0251 (17)	0.0230 (15)	0.0217 (16)	0.0059 (14)	0.0002 (14)	−0.0019 (12)
O8	0.0275 (18)	0.0218 (15)	0.0191 (17)	0.0046 (14)	0.0028 (14)	−0.0021 (12)
N1	0.0127 (16)	0.0199 (16)	0.0127 (15)	0.0003 (12)	−0.0018 (13)	−0.0023 (11)
N2	0.0167 (15)	0.0218 (14)	0.0135 (14)	0.0033 (18)	0.0023 (11)	−0.0025 (16)
N3	0.0147 (17)	0.0169 (14)	0.0143 (16)	0.0018 (12)	−0.0015 (13)	−0.0012 (12)
N4	0.0111 (15)	0.0173 (18)	0.0193 (16)	0.0017 (13)	0.0022 (13)	0.0000 (12)
C1	0.0047 (18)	0.0219 (19)	0.016 (2)	−0.0002 (15)	0.0014 (16)	0.0015 (15)
C2	0.0100 (17)	0.023 (2)	0.0151 (17)	−0.0005 (16)	0.0015 (14)	0.0015 (16)
C3	0.019 (2)	0.022 (2)	0.017 (2)	0.0030 (17)	0.0022 (18)	0.0007 (17)
C4	0.018 (2)	0.032 (2)	0.016 (2)	0.0010 (18)	−0.0011 (18)	0.0065 (17)
C5	0.013 (2)	0.032 (2)	0.020 (2)	−0.0014 (18)	0.0024 (18)	−0.0015 (18)
C6	0.018 (2)	0.0206 (19)	0.025 (2)	−0.0023 (17)	0.0010 (18)	−0.0026 (17)
C7	0.018 (2)	0.0207 (19)	0.016 (2)	−0.0005 (17)	−0.0019 (17)	0.0003 (17)
C8	0.030 (3)	0.037 (2)	0.020 (2)	0.004 (2)	0.0038 (19)	−0.0036 (18)
C9	0.032 (3)	0.037 (2)	0.017 (2)	−0.003 (2)	−0.0037 (19)	−0.0044 (18)
C10	0.012 (2)	0.025 (2)	0.013 (2)	0.0007 (16)	−0.0015 (16)	0.0025 (15)
C11	0.0102 (17)	0.025 (2)	0.0131 (16)	−0.0027 (18)	0.0017 (13)	−0.0030 (17)
C12	0.016 (2)	0.0215 (18)	0.018 (2)	−0.0017 (17)	0.0013 (17)	0.0015 (17)
C13	0.016 (2)	0.0208 (19)	0.024 (2)	−0.0016 (16)	0.0032 (18)	−0.0032 (16)
C14	0.012 (2)	0.030 (2)	0.015 (2)	−0.0048 (17)	0.0006 (17)	−0.0030 (17)
C15	0.023 (2)	0.027 (2)	0.017 (2)	−0.0028 (18)	0.0006 (19)	0.0028 (16)
C16	0.019 (2)	0.022 (2)	0.015 (2)	−0.0013 (17)	0.0012 (17)	−0.0003 (17)
C17	0.025 (2)	0.033 (2)	0.019 (2)	−0.0076 (19)	0.0046 (18)	−0.0087 (18)
C18	0.030 (3)	0.041 (3)	0.026 (2)	−0.005 (2)	−0.005 (2)	−0.010 (2)
C19	0.015 (2)	0.0157 (16)	0.0167 (19)	−0.0004 (15)	0.0004 (16)	0.0012 (15)
C20	0.014 (2)	0.0180 (18)	0.0182 (19)	0.0005 (15)	−0.0045 (16)	0.0027 (15)
C21	0.013 (2)	0.0201 (18)	0.020 (2)	0.0013 (16)	0.0022 (16)	0.0062 (16)
C22	0.0128 (17)	0.0175 (15)	0.0131 (16)	0.005 (2)	−0.0017 (13)	0.0017 (19)
C23	0.0154 (18)	0.021 (2)	0.0127 (17)	−0.0006 (16)	−0.0013 (14)	−0.0026 (15)
C24	0.011 (2)	0.023 (2)	0.022 (2)	0.0009 (16)	0.0042 (17)	0.0001 (16)
C25	0.026 (2)	0.0157 (18)	0.019 (2)	0.0060 (17)	0.0014 (17)	−0.0002 (15)
C26	0.030 (3)	0.0201 (19)	0.028 (2)	−0.0008 (18)	−0.001 (2)	0.0021 (16)
C27	0.016 (2)	0.028 (2)	0.020 (2)	0.0004 (16)	0.0063 (17)	−0.0047 (16)
C28	0.025 (2)	0.029 (2)	0.027 (2)	−0.0029 (19)	−0.0009 (19)	−0.0049 (19)
C29	0.019 (2)	0.0158 (18)	0.0143 (19)	0.0020 (15)	0.0001 (16)	−0.0024 (14)
C30	0.017 (2)	0.0174 (18)	0.017 (2)	−0.0024 (16)	−0.0003 (16)	−0.0006 (15)
C31	0.012 (2)	0.0177 (18)	0.025 (2)	−0.0021 (16)	0.0022 (17)	0.0027 (17)
C32	0.0130 (17)	0.0173 (19)	0.0150 (16)	0.0031 (18)	0.0007 (14)	0.0014 (16)
C33	0.0129 (18)	0.0157 (19)	0.0147 (17)	0.0031 (15)	−0.0041 (14)	−0.0009 (15)
C34	0.0103 (19)	0.0168 (18)	0.018 (2)	−0.0027 (15)	0.0011 (16)	0.0000 (15)
C35	0.019 (2)	0.028 (2)	0.018 (2)	0.0010 (17)	0.0047 (16)	−0.0030 (17)
C36	0.026 (3)	0.034 (2)	0.028 (2)	−0.006 (2)	0.002 (2)	−0.012 (2)
C37	0.020 (2)	0.0191 (19)	0.020 (2)	0.0014 (17)	0.0027 (17)	−0.0008 (15)
C38	0.037 (3)	0.0176 (18)	0.025 (2)	0.0008 (18)	0.001 (2)	0.0027 (17)

### Geometric parameters (Å, °)

**Table:**

Cu1—O1	1.968 (2)	C15—C16	1.395 (6)
Cu1—O3	1.979 (2)	C15—H15	0.9300
Cu1—O7	2.486 (3)	C16—H16	0.9300
Cu1—O8	2.439 (3)	C17—H17A	0.9700
Cu1—N1	2.004 (3)	C17—H17B	0.9700
Cu1—N3	2.004 (3)	C18—C17	1.511 (6)
O1—C1	1.275 (4)	C18—H18A	0.9600
O2—C1	1.253 (4)	C18—H18B	0.9600
O3—C10	1.268 (4)	C18—H18C	0.9600
O4—C10	1.234 (4)	C19—C20	1.384 (5)
O5—C24	1.224 (5)	C19—H19	0.9300
O6—C34	1.237 (4)	C20—H20	0.9300
O7—H71	0.851 (19)	C21—C20	1.386 (5)
O7—H72	0.839 (19)	C21—H21	0.9300
O8—H81	0.854 (19)	C22—C21	1.400 (6)
O8—H82	0.859 (19)	C22—C23	1.382 (5)
N1—C19	1.347 (5)	C22—C24	1.493 (5)
N1—C23	1.338 (4)	C23—H23	0.9300
N2—C24	1.365 (5)	C25—C26	1.520 (6)
N2—C25	1.459 (5)	C25—H25A	0.9700
N2—C27	1.463 (5)	C25—H25B	0.9700
N3—C29	1.338 (5)	C26—H26A	0.9600
N3—C33	1.340 (4)	C26—H26B	0.9600
N4—C34	1.346 (5)	C26—H26C	0.9600
N4—C35	1.459 (5)	C27—H27A	0.9700
C2—C1	1.495 (5)	C27—H27B	0.9700
C2—C3	1.394 (5)	C28—C27	1.520 (6)
C3—H3	0.9300	C28—H28A	0.9600
C4—C3	1.394 (6)	C28—H28B	0.9600
C4—C5	1.383 (6)	C28—H28C	0.9600
C4—H4	0.9300	C29—H29	0.9300
C5—C6	1.381 (6)	C30—C29	1.383 (5)
C5—C8	1.526 (6)	C30—C31	1.383 (5)
C6—H6	0.9300	C30—H30	0.9300
C7—C2	1.394 (6)	C31—H31	0.9300
C7—C6	1.382 (5)	C32—C31	1.396 (6)
C7—H7	0.9300	C32—C33	1.384 (5)
C8—C9	1.519 (6)	C32—C34	1.495 (5)
C8—H8A	0.9700	C33—H33	0.9300
C8—H8B	0.9700	C35—C36	1.517 (5)
C9—H9A	0.9600	C35—H35A	0.9700
C9—H9B	0.9600	C35—H35B	0.9700
C9—H9C	0.9600	C36—H36A	0.9600
C11—C10	1.513 (5)	C36—H36B	0.9600
C11—C12	1.385 (6)	C36—H36C	0.9600
C11—C16	1.384 (5)	C37—N4	1.466 (5)
C12—H12	0.9300	C37—C38	1.520 (6)
C13—C12	1.384 (5)	C37—H37A	0.9700
C13—C14	1.379 (6)	C37—H37B	0.9700
C13—H13	0.9300	C38—H38A	0.9600
C14—C17	1.512 (5)	C38—H38B	0.9600
C15—C14	1.390 (5)	C38—H38C	0.9600
			
O1—Cu1—O3	179.27 (14)	C17—C18—H18B	109.5
O1—Cu1—O8	88.46 (11)	C17—C18—H18C	109.5
O1—Cu1—N1	89.89 (11)	H18A—C18—H18B	109.5
O1—Cu1—N3	88.39 (11)	H18A—C18—H18C	109.5
O3—Cu1—O8	92.16 (11)	H18B—C18—H18C	109.5
O3—Cu1—N1	89.77 (11)	N1—C19—C20	122.3 (3)
O3—Cu1—N3	91.93 (12)	N1—C19—H19	118.8
N1—Cu1—O8	86.48 (11)	C20—C19—H19	118.8
N3—Cu1—O8	95.55 (11)	C19—C20—C21	118.9 (4)
N3—Cu1—N1	177.30 (13)	C19—C20—H20	120.6
C1—O1—Cu1	128.0 (3)	C21—C20—H20	120.6
C10—O3—Cu1	128.3 (3)	C20—C21—C22	119.4 (4)
H71—O7—H72	101 (5)	C20—C21—H21	120.3
Cu1—O8—H81	90 (3)	C22—C21—H21	120.3
Cu1—O8—H82	137 (3)	C21—C22—C24	123.5 (3)
H81—O8—H82	100 (4)	C23—C22—C21	117.5 (3)
C19—N1—Cu1	120.8 (2)	C23—C22—C24	118.9 (4)
C23—N1—Cu1	120.9 (2)	N1—C23—C22	123.7 (3)
C23—N1—C19	118.2 (3)	N1—C23—H23	118.2
C24—N2—C25	123.9 (3)	C22—C23—H23	118.2
C24—N2—C27	117.6 (4)	O5—C24—N2	123.4 (4)
C25—N2—C27	117.6 (3)	O5—C24—C22	119.9 (4)
C29—N3—Cu1	121.3 (3)	N2—C24—C22	116.8 (3)
C29—N3—C33	118.5 (3)	N2—C25—C26	112.9 (3)
C33—N3—Cu1	120.1 (2)	N2—C25—H25A	109.0
C34—N4—C35	119.4 (3)	N2—C25—H25B	109.0
C34—N4—C37	123.4 (3)	C26—C25—H25A	109.0
C35—N4—C37	117.2 (3)	C26—C25—H25B	109.0
O1—C1—C2	116.0 (3)	H25A—C25—H25B	107.8
O2—C1—O1	124.6 (4)	C25—C26—H26A	109.5
O2—C1—C2	119.4 (3)	C25—C26—H26B	109.5
C3—C2—C1	120.9 (4)	C25—C26—H26C	109.5
C3—C2—C7	118.8 (3)	H26A—C26—H26B	109.5
C7—C2—C1	120.2 (3)	H26A—C26—H26C	109.5
C2—C3—C4	119.5 (4)	H26B—C26—H26C	109.5
C2—C3—H3	120.3	N2—C27—C28	111.6 (3)
C4—C3—H3	120.3	N2—C27—H27A	109.3
C3—C4—H4	119.4	N2—C27—H27B	109.3
C5—C4—C3	121.2 (4)	C28—C27—H27A	109.3
C5—C4—H4	119.4	C28—C27—H27B	109.3
C4—C5—C8	120.5 (5)	H27A—C27—H27B	108.0
C6—C5—C4	119.1 (4)	C27—C28—H28A	109.5
C6—C5—C8	120.3 (4)	C27—C28—H28B	109.5
C5—C6—C7	120.3 (4)	C27—C28—H28C	109.5
C5—C6—H6	119.8	H28A—C28—H28B	109.5
C7—C6—H6	119.8	H28A—C28—H28C	109.5
C2—C7—H7	119.5	H28B—C28—H28C	109.5
C6—C7—C2	121.0 (4)	N3—C29—C30	122.5 (3)
C6—C7—H7	119.5	N3—C29—H29	118.7
C5—C8—H8A	109.3	C30—C29—H29	118.7
C5—C8—H8B	109.3	C29—C30—H30	120.5
C9—C8—C5	111.7 (4)	C31—C30—C29	119.0 (4)
C9—C8—H8A	109.3	C31—C30—H30	120.5
C9—C8—H8B	109.3	C30—C31—C32	118.9 (4)
H8A—C8—H8B	107.9	C30—C31—H31	120.5
C8—C9—H9A	109.5	C32—C31—H31	120.5
C8—C9—H9B	109.5	C31—C32—C34	123.4 (3)
C8—C9—H9C	109.5	C33—C32—C31	118.2 (3)
H9A—C9—H9B	109.5	C33—C32—C34	118.2 (4)
H9A—C9—H9C	109.5	N3—C33—C32	122.8 (3)
H9B—C9—H9C	109.5	N3—C33—H33	118.6
O3—C10—C11	115.5 (3)	C32—C33—H33	118.6
O4—C10—O3	126.3 (4)	O6—C34—N4	122.2 (4)
O4—C10—C11	118.2 (4)	O6—C34—C32	119.2 (4)
C12—C11—C10	121.3 (4)	N4—C34—C32	118.6 (3)
C16—C11—C10	119.6 (4)	N4—C35—C36	112.2 (3)
C16—C11—C12	119.0 (3)	N4—C35—H35A	109.2
C11—C12—H12	119.9	N4—C35—H35B	109.2
C13—C12—C11	120.1 (4)	C36—C35—H35A	109.2
C13—C12—H12	119.9	C36—C35—H35B	109.2
C12—C13—H13	119.3	H35A—C35—H35B	107.9
C14—C13—C12	121.5 (4)	C35—C36—H36A	109.5
C14—C13—H13	119.3	C35—C36—H36B	109.5
C13—C14—C15	118.5 (4)	C35—C36—H36C	109.5
C13—C14—C17	120.2 (4)	H36A—C36—H36B	109.5
C15—C14—C17	121.3 (4)	H36A—C36—H36C	109.5
C14—C15—C16	120.3 (4)	H36B—C36—H36C	109.5
C14—C15—H15	119.8	N4—C37—C38	112.4 (3)
C16—C15—H15	119.8	N4—C37—H37A	109.1
C11—C16—C15	120.5 (4)	N4—C37—H37B	109.1
C11—C16—H16	119.7	C38—C37—H37A	109.1
C15—C16—H16	119.7	C38—C37—H37B	109.1
C14—C17—H17A	109.2	H37A—C37—H37B	107.9
C14—C17—H17B	109.2	C37—C38—H38A	109.5
C18—C17—C14	112.2 (4)	C37—C38—H38B	109.5
C18—C17—H17A	109.2	C37—C38—H38C	109.5
C18—C17—H17B	109.2	H38A—C38—H38B	109.5
H17A—C17—H17B	107.9	H38A—C38—H38C	109.5
C17—C18—H18A	109.5	H38B—C38—H38C	109.5
			
O8—Cu1—O1—C1	−150.2 (3)	C7—C2—C3—C4	1.5 (6)
N1—Cu1—O1—C1	123.4 (3)	C5—C4—C3—C2	−0.1 (7)
N3—Cu1—O1—C1	−54.6 (3)	C3—C4—C5—C6	−1.6 (8)
O8—Cu1—O3—C10	−27.2 (3)	C3—C4—C5—C8	176.3 (4)
N1—Cu1—O3—C10	59.3 (3)	C4—C5—C6—C7	1.8 (7)
N3—Cu1—O3—C10	−122.8 (3)	C8—C5—C6—C7	−176.1 (4)
O1—Cu1—N1—C19	39.5 (3)	C4—C5—C8—C9	−86.2 (6)
O1—Cu1—N1—C23	−143.5 (3)	C6—C5—C8—C9	91.7 (5)
O3—Cu1—N1—C19	−141.1 (3)	C6—C7—C2—C1	−179.3 (4)
O3—Cu1—N1—C23	35.8 (3)	C6—C7—C2—C3	−1.3 (6)
O8—Cu1—N1—C19	−48.9 (3)	C2—C7—C6—C5	−0.4 (6)
O8—Cu1—N1—C23	128.0 (3)	C12—C11—C10—O3	3.4 (5)
O1—Cu1—N3—C29	134.7 (3)	C12—C11—C10—O4	−175.6 (4)
O1—Cu1—N3—C33	−40.3 (3)	C16—C11—C10—O3	−178.6 (3)
O3—Cu1—N3—C29	−44.7 (3)	C16—C11—C10—O4	2.4 (6)
O3—Cu1—N3—C33	140.3 (3)	C10—C11—C12—C13	178.2 (4)
O8—Cu1—N3—C29	−137.0 (3)	C16—C11—C12—C13	0.2 (6)
O8—Cu1—N3—C33	48.0 (3)	C10—C11—C16—C15	−179.3 (4)
Cu1—O1—C1—O2	−27.7 (5)	C12—C11—C16—C15	−1.3 (6)
Cu1—O1—C1—C2	152.8 (2)	C14—C13—C12—C11	1.4 (6)
Cu1—O3—C10—O4	28.2 (6)	C12—C13—C14—C15	−2.0 (7)
Cu1—O3—C10—C11	−150.8 (2)	C12—C13—C14—C17	176.4 (4)
Cu1—N1—C23—C22	−175.6 (3)	C13—C14—C17—C18	−73.7 (6)
C19—N1—C23—C22	1.4 (5)	C15—C14—C17—C18	104.7 (6)
Cu1—N1—C19—C20	177.7 (3)	C16—C15—C14—C13	1.0 (8)
C23—N1—C19—C20	0.7 (5)	C16—C15—C14—C17	−177.4 (4)
C25—N2—C24—O5	−168.2 (4)	C14—C15—C16—C11	0.7 (7)
C25—N2—C24—C22	11.8 (5)	N1—C19—C20—C21	−2.8 (6)
C27—N2—C24—O5	0.8 (6)	C22—C21—C20—C19	2.9 (6)
C27—N2—C24—C22	−179.2 (3)	C23—C22—C21—C20	−1.0 (6)
C24—N2—C25—C26	−111.3 (4)	C24—C22—C21—C20	177.1 (4)
C27—N2—C25—C26	79.6 (4)	C21—C22—C23—N1	−1.3 (6)
C24—N2—C27—C28	−90.0 (4)	C24—C22—C23—N1	−179.4 (3)
C25—N2—C27—C28	79.7 (4)	C21—C22—C24—O5	−109.8 (5)
Cu1—N3—C29—C30	−174.7 (3)	C21—C22—C24—N2	70.3 (5)
C33—N3—C29—C30	0.4 (5)	C23—C22—C24—O5	68.3 (5)
Cu1—N3—C33—C32	174.3 (3)	C23—C22—C24—N2	−111.7 (4)
C29—N3—C33—C32	−0.9 (5)	C31—C30—C29—N3	0.7 (6)
C35—N4—C34—O6	−1.9 (6)	C29—C30—C31—C32	−1.5 (6)
C35—N4—C34—C32	178.7 (3)	C33—C32—C31—C30	1.0 (6)
C37—N4—C34—O6	175.4 (4)	C34—C32—C31—C30	−173.7 (4)
C37—N4—C34—C32	−4.0 (5)	C31—C32—C33—N3	0.2 (5)
C34—N4—C35—C36	94.1 (4)	C34—C32—C33—N3	175.2 (3)
C37—N4—C35—C36	−83.4 (4)	C31—C32—C34—O6	100.3 (5)
C3—C2—C1—O1	176.3 (3)	C31—C32—C34—N4	−80.3 (5)
C3—C2—C1—O2	−3.3 (6)	C33—C32—C34—O6	−74.4 (5)
C7—C2—C1—O1	−5.8 (5)	C33—C32—C34—N4	105.0 (4)
C7—C2—C1—O2	174.7 (4)	C38—C37—N4—C34	104.0 (4)
C1—C2—C3—C4	179.5 (4)	C38—C37—N4—C35	−78.6 (4)

### Hydrogen-bond geometry (Å, °)

**Table:**

*D*—H···*A*	*D*—H	H···*A*	*D*···*A*	*D*—H···*A*
O7—H71···O2	0.85 (2)	1.86 (2)	2.706 (4)	170 (5)
O7—H72···O6^i^	0.83 (4)	2.03 (4)	2.846 (4)	166 (5)
O8—H81···O4	0.85 (2)	1.88 (2)	2.699 (4)	161 (5)
O8—H82···O2^ii^	0.86 (4)	2.00 (4)	2.852 (4)	167 (5)
C6—H6···O5^iii^	0.93	2.55	3.240 (5)	131
C20—H20···O2^iii^	0.93	2.53	3.412 (5)	158
C30—H30···O6^iv^	0.93	2.43	3.316 (5)	158

Symmetry codes: (i) −*x*+2, *y*+1/2, −*z*; (ii) −*x*+2, *y*−1/2, −*z*; (iii) −*x*+1, *y*−1/2, −*z*; (iv) −*x*+3, *y*+1/2, −*z*.

## References

[bb1] Allen, F. H., Kennard, O., Watson, D. G., Brammer, L., Orpen, A. G. & Taylor, R. (1987). *J. Chem. Soc. Perkin Trans. 2*, pp. S1–19.

[bb2] Bigoli, F., Braibanti, A., Pellinghelli, M. A. & Tiripicchio, A. (1972). *Acta Cryst.* B**28**, 962–966.

[bb3] Bruker (2007). *APEX2* and *SAINT* Bruker AXS Inc. Madison, Wisconsin, USA.

[bb4] Farrugia, L. J. (1997). *J. Appl. Cryst.* **30**, 565.

[bb5] Farrugia, L. J. (1999). *J. Appl. Cryst.* **32**, 837–838.

[bb6] Flack, H. D. (1983). *Acta Cryst.* A**39**, 876–881.

[bb7] Hökelek, T., Dal, H., Tercan, B., Özbek, F. E. & Necefoğlu, H. (2009*a*). *Acta Cryst.* E**65**, m466–m467. 10.1107/S1600536809011209 PMC2968958 21582397

[bb8] Hökelek, T., Dal, H., Tercan, B., Özbek, F. E. & Necefoğlu, H. (2009*b*). *Acta Cryst.* E**65**, m607–m608. 10.1107/S1600536809015645 PMC2977639 21583825

[bb9] Hökelek, T., Gündüz, H. & Necefoğlu, H. (1996). *Acta Cryst.* C**52**, 2470–2473.

[bb10] Hökelek, T. & Necefoğlu, H. (1998). *Acta Cryst.* C**54**, 1242–1244.

[bb11] Hökelek, T. & Necefoğlu, H. (2007). *Acta Cryst.* E**63**, m821–m823.

[bb12] Krishnamachari, K. A. V. R. (1974). *Am. J. Clin. Nutr.* **27**, 108–111. 10.1093/ajcn/27.2.108 4812927

[bb13] Necefoğlu, H., Maracı, A., Özbek, F. E., Tercan, B. & Hökelek, T. (2011). *Acta Cryst.* E**67**, m619–m620. 10.1107/S1600536811014188 PMC3089307 21754332

[bb14] Sheldrick, G. M. (2008). *Acta Cryst.* A**64**, 112–122. 10.1107/S0108767307043930 18156677

[bb15] Spek, A. L. (2009). *Acta Cryst.* D**65**, 148–155. 10.1107/S090744490804362X PMC2631630 19171970

